# Rats prefer mutual rewards in a prosocial choice task

**DOI:** 10.3389/fnins.2014.00443

**Published:** 2015-01-15

**Authors:** Julen Hernandez-Lallement, Marijn van Wingerden, Christine Marx, Milan Srejic, Tobias Kalenscher

**Affiliations:** Comparative Psychology, Institute of Experimental Psychology, Heinrich-Heine University of DüsseldorfDüsseldorf, Germany

**Keywords:** social behavior, prosocial behavior, decision making, rats, prosocial choice task

## Abstract

Pro-sociality, i.e., the preference for outcomes that produce benefits for other individuals, is ubiquitous in humans. Recently, cross-species comparisons of social behavior have offered important new insights into the evolution of pro-sociality. Here, we present a rodent analog of the Pro-social Choice Task that controls strategic components, de-confounds other-regarding choice motives from the animals' natural tendencies to maximize own food access and directly tests the effect of social context on choice allocation. We trained pairs of rats—an actor and a partner rat—in a double T-maze task where actors decided between two alternatives only differing in the reward delivered to the partner. The “own reward” choice yielded a reward only accessible to the actor whereas the “both reward” choice produced an additional reward for a partner (partner condition) or an inanimate toy (toy Condition), located in an adjacent compartment. We found that actors chose “both reward” at levels above chance and more often in the partner than in the toy condition. Moreover, we show that this choice pattern adapts to the current social context and that the observed behavior is stable over time.

## Introduction

Classic economic theory posits that decisions should be exclusively motivated by self-interest, and decisions makers should therefore disregard other individuals' needs (Von Neumann and Morgenstern, 1994; Fehr and Schmidt, 2006). However, empirical evidence does not support this prediction and rather suggests that people actively and spontaneously share acquired goods (Koch, 2008; Muehlbacher and Kirchler, 2009; Hernandez-Lallement et al., 2013) and care about others (Bernhard et al., 2006). Furthermore, people are adept in detecting and responding to unfairness (Sanfey et al., 2003) and inequity (Sanfey, 2007), and engage in costly behaviors to punish social norm-violation and enforce social norm compliance (De Quervain et al., 2004).

Such behaviors are not just restricted to humans but can be found throughout the animal world, from social choice in our close primate relatives (Burkart et al., 2007; Yamamoto et al., 2009; Cronin et al., 2010; Horner et al., 2011) to the eusocial communities of the ants (Nowbahari et al., 2009). Although the non-human primate models yield important insights into the evolutionary roots of pro-sociality (Brosnan and De Waal, 2003; Cronin, 2012) and their neural underpinnings (Chang et al., 2013), other animals such as rats might offer an equally powerful model to investigate the evolution and neural substrates of social behavior (Kim et al., 2010; Atsak et al., 2011; Kashtelyan et al., 2014; Willuhn et al., 2014). Rats are ideally suited to study social choice behavior. For instance, rats often develop in social groups (Whishaw and Kolb, 2005), have clear hierarchical group organization (Baenninger, 1966) and prefer to eat close to conspecifics (Barnett and Spencer, 1951). Moreover, they are able to display cooperative coordination (Schuster, 2002) as well as direct (Rutte and Taborsky, 2007a) and generalized reciprocity (Pfeiffer et al., 2005; Rutte and Taborsky, 2007b). Furthermore, it has been suggested that helping behavior might selectively be engaged depending on the state and bodily mass of a partner (Schneeberger et al., 2012), suggesting that social interaction patterns in rats are dynamic (Ben-Ami Bartal et al., 2014). Finally, it has been recently suggested that rats feel empathy (Ben-Ami Bartal et al., 2011; but see Silberberg et al., 2014). Thus, given that rats are capable of engaging in behaviors that produce benefits for conspecifics, this specie is ideally suited to study the evolution, psychology and neuroscience of social behavior.

Hence, what is needed is a standardized, simple, fast and easy-to-train social choice task for rodents. This task should eliminate strategic, reciprocal or other egoistic motivational components and make the underlying cognitive choice mechanisms tractable. Moreover, a sound design should involve non-costly choices to de-confound pro-social motives from the animals' natural tendencies to maximize own-access to food as strong egoistic motives may compete, and thus obscure, pro-social sentiments (Silk et al., 2005). Insights gained from such a standardized animal model will facilitate cross-species comparison of pro-social behavior and will shed light on common evolutionary roots and factors favoring pro-social behavior (Dugatkin, 1997; Kalenscher and van Wingerden, 2011; Brosnan and de Waal, 2014). Finally, a good paradigm should allow the full range of neurobiological manipulations, including behavioral, pharmacological and electrophysiological measurements, paving the path for manipulation and recording of neural activity during social decision making to enhance understanding and modeling of decision making in social contexts.

The scope of this study is to introduce a rodent analog of the Prosocial Choice Task (PCT; Silk et al., 2005; Marquez and Moita, 2012), a simple and standardized behavioral experimental paradigm adapted from a well-established task in primates (Burkart et al., 2007; Horner et al., 2011), to probe pro-social choice behavior. In line with definitions used in the literature (Miller et al., 1991), we define pro-social choice by its face validity as the preference for outcomes that produce a benefit for another individual. We hypothesized that rats behave pro-socially according to the above definition. In this task, rats (*hereafter: actors*) had to choose between two options yielding either only a reward for themselves (“own reward” OR; 1/0) or an additional reward to a partner (“both reward” BR; 1/1). Crucially, we compared the actors' BR preferences in a partner condition, in which the partner was an actual rat, with its BR preferences in a toy condition, where the partner was an inanimate toy rat of similar shape, size and color. We conjectured that, if a conspecific's access to food carries reinforcing value for actor rats (Kashtelyan et al., 2014), they should develop a preference for the “both-reward” alternative in the partner, but not the toy condition. Our main results confirmed this hypothesis: actors chose “both reward” at levels above chance and more often when paired with another rat than with an inanimate toy, suggesting that BR-preferences were dependent on social components of the task. Interestingly, there were large individual differences in the rats' propensity to choose the “both-reward” alternative, which might rely on difference in partner's body weight. Finally, we show that the rats' social-context-dependent preferences for the BR alternative remained stable after a repetition manipulation, suggesting that social preferences are stable over time.

## Materials and methods

### Subjects and housing

Two batches (*N* = 20 and *N* = 48, respectively) of male Long-Evans rats (*Janvier Labs, St. Berthevin, France*) were used (See Supplementary Data). Animals were housed in groups of four rats per cage. In a study addressing the effect of food deprivation on choice in social context in rats, higher cooperation rates have been observed in sated rats compared to food deprived rats (Viana et al., 2010). Additionally, recent findings suggest that cooperation rates are influenced by multiple factors, including body weight (Schneeberger et al., 2012). Thus, we opted for a merely mild food deprivation schedule, and daily food intake was restricted to keep animals at >85% of free feeding body weight; to monitor the effect of body weight on social behavior, we included weight as a factor in our analyses to identify its putatively mediating effect on choice allocation (see below). Water was available *ad libitum* in the home cage. All animal procedures adhered to the German Welfare Act and were approved by the LANUV (Landesamt für Natur-, Umwelt- und Verbaucherschutz North Rhine-Westphalia, Germany).

### Experimental setup

**Figure 1:**
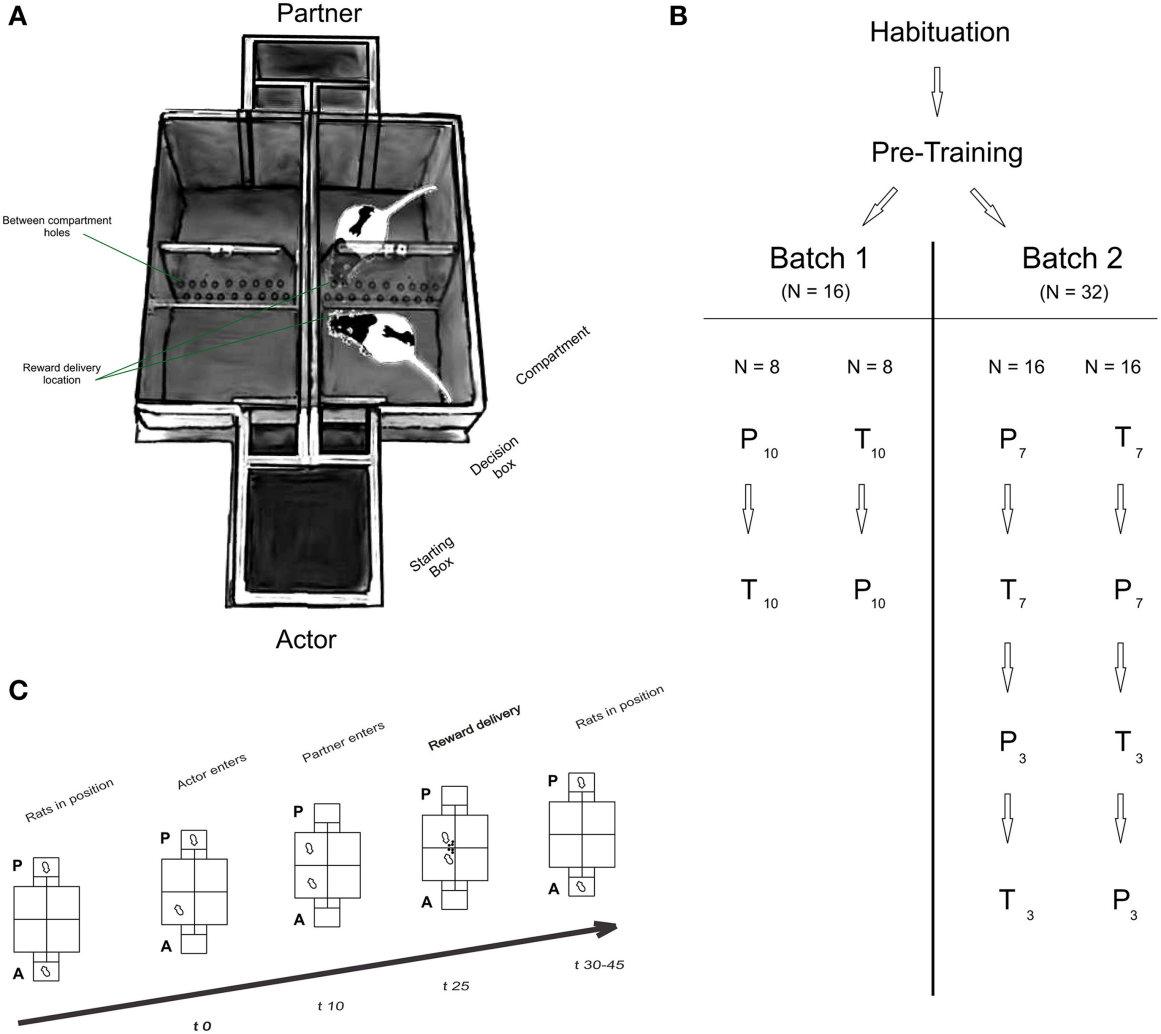
**(A)** Experimental setup, **(B)** organization of groups and batches and **(C)** timeline of a typical trial. **(A)** Apparatus: each T Maze consisted of a starting box equipped with two independent doors that led to a decision box. A second door gave access to either compartment. Perforated and transparent walls were placed between compartments and between T Mazes to allow olfactory, auditory and visual communication between rats. A semi-automatic reward delivery system was placed at the intersection of each perforated wall, i.e., at the center of the maze (not shown on figure). **(B)** Organization of groups and batches of rats: all rats underwent habituation and pre-training procedures (See Supplementary Data). Actors in batch 1 (*N* = 16) and batch 2 (*N* = 32) were split in two groups, each starting in either the partner (P) or toy (T) condition. Each condition consisted of ten consecutive sessions in batch 1 (indicated by the subscript 10), or 7 sessions in batch 2 (pre-repetition), followed by three post-repetition sessions. **(C)** Structure of a typical trial: The actor **(A)** always moved first into one of the two compartments (in free-choice trials), or into only one compartment (in forced-choice trials; time 0 s; *t0*; trial onset). 10 s later (*t10*), the partner rat was directed into the compartment facing the compartment chosen by the actor. In the toy condition, the experimenter manually placed the toy in the respective compartment. 25 s after trial onset (*t25*) rewards were delivered to the actor only (after own-reward (OR) choices) or both rats (after both-reward (BR) choices). After reward consumption, rats were placed back in their start box positions, and a new trial started after a variable inter-trial interval (ITI; *t30-45*).

Experiments were conducted in a custom-made double T-Maze (Figure 1A), with the mazes' main compartments facing each other. The T-mazes were separated by a transparent and perforated wall allowing olfactory, auditory and visual communication. Each T-maze consisted of a starting box connected to two decision chambers by two independently operated doors, each leading to a choice compartment. To prevent the experimenters operating the setups from influencing the rats' behavior, compartments and starting boxes were constantly covered using removable red tops. The data from the first batch of rats was not collected using covers. Rewards (45 mg dustless precision pellets, Bio-Serv, Germany), delivered in the inner corner of the compartments through a funnel system, were hidden from the animals during the decision phase, thus minimizing potential distractive or competitive motives (see Cronin, 2012 for an extensive discussion of this point).

### Experimental design

During the whole duration of the experiment, every actor was trained for one session a day on five consecutive weekdays for all habituation, training (see Supplementary Data) and testing sessions.

#### Groups and batches

The general structure of the experiment is described in Figure 1B. In batch 1, four rats from the same cage were assigned to the “*partner”* group and 16 rats were assigned to the “*actor*” group. In batch 2, 16 rats were used as partners and 32 animals were used as actors.

Actor and partner rats were never housed together. The actors were tested for four consecutive weeks paired with either a partner (partner condition) or a toy rat (toy condition), depending on the testing condition. Actors were always paired with the same partner.

#### General task design

Rats were tested in two main conditions: in the partner condition, both actor and partner rats were placed in the maze in their respective starting boxes; in the toy condition, a toy rat was used as partner. The actor always moved first and could enter either compartment. The partner never had a choice, i.e., the experimenter always directed the partner to the compartment facing the actor. After entering either compartment, actors received an identical amount of reward (three sucrose pellets), delivered after the same delay. Importantly, entering one compartment (“both reward—BR” compartment) resulted in a reward delivery of same magnitude and delay in both the actors' and partner's compartments, whereas deciding for the alternative choice (the “own reward—OR” compartment) resulted in reward delivery to the actor rat compartment only.

In the toy condition, the partner was an inanimate toy rat of similar size, shape and color. The toy condition served as a control for pellet delivery sounds and potential secondary reinforcement effects of the food delivery. Importantly, the choice-reward payoff structure was identical across partner and toy conditions, i.e., rewards were delivered to the toy rat compartment with the same magnitude and delay as in the partner condition. Thus, any difference in choice allocation between the partner and the toy conditions could be attributed to the influence of social context on the actor's decisions. Note that a significant preference for the BR- or OR-alternative would suggest that the rats have some knowledge of the task structure, but it would not allow us to make inferences about the precise nature of their knowledge.

#### Typical trial structure

Each trial followed a strict time schedule (Figure 1C) to guarantee invariant response times and reward delays. By doing so, we ensured that the actors' preferences for one compartment were not merely the results of asymmetrically timed reward deliveries. Both rats were placed in the respective starting boxes of the maze at the beginning of the session (Figure 1C; Baseline). The experimenter opened the actor's door and waited for the animal to enter one of the compartments. Door opening marked trial onset. For rats in batch 1, the rats had 10 s to enter the compartment. Once a rat had fully entered a compartment with all four paws, the door was closed, “trapping” the rat inside the compartment (Figure 1C; Trial start, *t0*). Ten seconds later, the partner (or toy) was directed (by opening only one door) or placed (the toy was manually placed) by the experimenter into the compartment facing the actor rat's compartment (Figure 1C; Partner enters, *t10*). Occasionally, partner rats were slow to enter the compartment, in which case the experimenter gently pushed the rat into the compartment, making sure that the strict time schedule was met. Reward was always delivered 25 s after trial onset (Figure 1C; Reward delivery, *t25*) simultaneously into both compartments (after BR choices) or to the actor's compartment only (OR choices). After reward consumption, rats were manually replaced in their respective starting boxes to start a new trial. In the toy condition, the experimenter then removed the pellets delivered to the toy. The inter-trial interval (Figure 1C; ITI, *t30-45*) duration was independent of the actor's choice.

#### Test sessions

A session began with 10 (Batch 1) or eight (Batch 2) forced-choice trials (actors were forced to enter either compartment in a pseudo-randomized order) in order to allow sampling of the compartment / outcome contingencies. The forced-choice trials were followed by 15 free choice trials in which the actors could freely choose which compartment to enter. Each rat was tested in 10 (batch 1) or seven (batch 2; see next paragraph) consecutive sessions in the partner-condition, and the same amount of additional sessions in the toy condition. To control for potential order effects, half of the actors started testing in the partner condition, followed by the toy condition, with the reverse order for the other half of rats. We found no order effect on rats' between-condition preferences (See Supplementary Data).

To probe stability of preferences over time and social contexts, animals in batch 2 were tested in seven sessions (Pre-Repetition) in the partner condition, followed by the toy conditions (or vice versa), and were subsequently retested for three sessions in each condition again (Post-Repetition), thus amounting to a total number of 10 sessions per condition.

To control for potential side biases, left and right compartments were pseudo-randomly assigned as BR or OR compartments within rats and across sessions; thus, BR and OR sides differed within and across rats and testing days. Moreover, all experiments were carried out in red light in a closed black curtain system, to minimize the influence of contextual cues on decision making. Throughout the experiment, the experimenter was positioned at the end of the maze during decision process and reward consumption. To prevent rats from moving toward or away from the experimenter, and thus creating an artificial side bias, the experimenter moved between trials, independently of the BR or OR side allocation (see Supplementary Data). To control for social exploration motives, systematic approach/avoidance behavior as well as possible effects of closeness while eating (Barnett and Spencer, 1951), the partner was always directed into the compartment directly facing the compartment chosen by the actor, thus keeping the average distance between animals after entering the choice compartments equal and independent of the actors' choices.

### Analysis

#### Social bias

In addition to recording the percentage of BR choices relative to all choices, we calculate, for each rat, a social bias score (SB). The social bias score for rat *i* was expressed as the percent change in absolute BR choices in the partner condition [BR(partner)_i_] relative to the BR choices in the toy condition [BR(toy)_i_]:

(1) $$SB_{i}=\left[\frac{\text{BR}{\left(\text{partner}\right)}_{i}-\text{BR}{\left(\text{toy}\right)}_{i}}{\text{BR}{\left(\text{toy}\right)}_{i}}\right]*100$$

Positive and negative SB-values quantify the tendency to choose the BR compartment more or less often in the partner condition relative to the toy condition.

#### Weight analysis

We related the actors' propensities to make BR choices to their body weights. Because rats in the two batches had different body masses, in order to establish commensurability between batches, the mean weights of each actor *i* were normalized to their initial weight in the first session using the following equation:

(2) $$NormWeight_{i}=\left[\frac{MeanWeight_{i}-Sess1Weight_{i}}{Sess1Weight_{i}}\right]*100$$

## Results

We analyzed the actor rats' choice allocations in the two batches (Figure 1B; *N* = 16 and *N* = 32, respectively) separately because of differences in the experimental design (See Materials and Methods). All rats completed all trials and sessions.

### Actor rats have a preference for the BR compartment when paired with a partner rat

**Figure 2:**
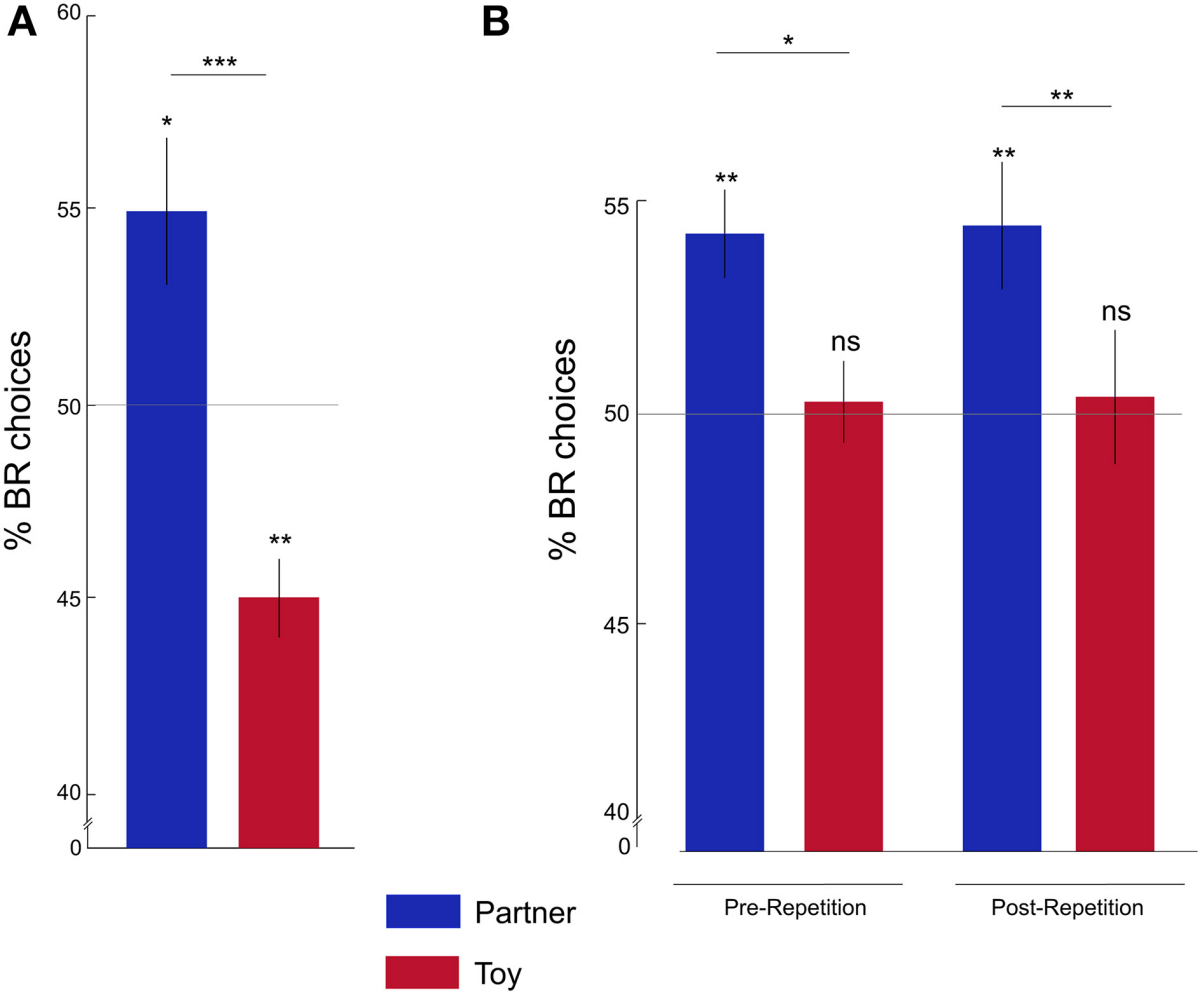
**Rats show pro-social behavior. (A)** Percentage of BR choices for the partner (blue) and toy (red) conditions in Batch 1: the average percentage of BR choices was significantly higher in the partner compared to the toy condition and was different from chance levels. **(B)** Percentage of BR choices in Batch 2: rats showed the same partner-toy-dissociation of pro-social behavior pre- and post-repetition. Y-axis is cut for demonstration purposes. ^*^p < 0.05; ^**^p < 0.01; ^***^p < 0.001; ns, not significant. Error bars represent the standard error of the mean, s.e.m.

We first asked whether, at the group level, rats' preferences for BR or OR compartments were significantly different from chance, and, further, whether their preferences differed between partner and toy conditions. In batch 1 (see below for batch 2 results), the proportion of BR choices was significantly above chance in the partner condition (One-sample Wilcoxon signed rank test; *Z* = 2.54; *p* = 0.01), and significantly below chance in the toy condition (*Z* = −2.95; *p* = 0.003). Accordingly, we found a significantly higher proportion of BR choices in the partner condition compared to the toy condition (Figure 2A; Wilcoxon matched-pairs signed rank test *Z* = −3.41; *p* = 0.001).

### Choice preferences are stable over time and faster re-acquired after repetition

To investigate whether the individual choice allocation pattern was stable over time, we tested the second batch of rats for seven sessions per condition, and then re-tested them in a repetition phase of three sessions per condition, thus repeatedly and successively alternating between partner and toy conditions (Figure 2B). The percentage of BR choices was significantly higher than chance for both Pre- and Post-repetition in the partner condition ([Pre] *Z* = 3.32; *p* = 0.001; [Post] *Z* = 2.66; *p* = 0.008), but not in the toy condition ([Pre] *Z* = −0.11; *p* = 0.91; [Post] *Z* = 0.10; *p* = 0.92).

Moreover, we found a significantly higher percentage of BR choices in the partner compared to the toy condition in the Pre- (*Z* = −2.14; *p* = 0.03) and Post-repetition (*Z* = −2.42; *p* = 0.01) phases. The percentage of BR choices did not significantly differ between Pre- and Post-repetition in either condition ([Partner] *Z* = −0.17; *p* = 0.87; [Toy] *Z* = −0.13; *p* = 0.90). These results suggest that choice preferences were stable over time.

### Individual differences in pro-social behavior

**Figure 3:**
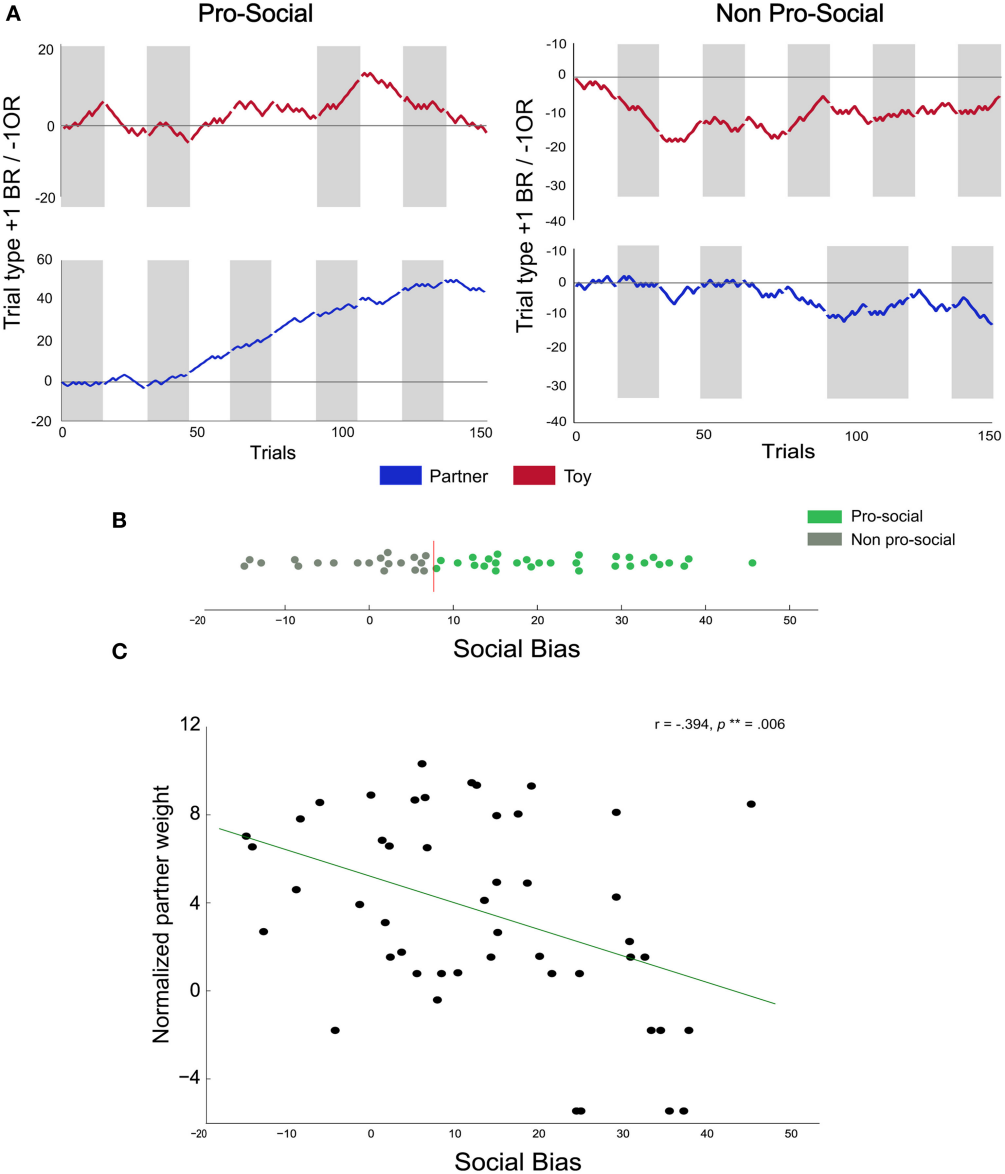
**Individual differences in pro-social behavior. (A)** Cumulative choice plots illustrate individual differences in pro-social behavior: for each trial, the running pro-social tally is incremented by +1 for each BR choice, and decremented by −1 for each OR choice. Thus, a monotonous upward slope indicates consistent BR choices across trials and sessions, neutral slopes indicate indifference, and negative slopes indicate consistent OR choices. Gray areas represent sessions where the BR compartment was on the left side. The two left panels show the cumulative choice plots of a rat classified as pro-social (performance in the toy condition indicated in red, upper left panel, performance in the partner condition indicated in blue in the 2nd down left panel). The two right panels show the choice data from a rat classified as non-pro-social. **(B)** Social bias scores of all rats: colors represent rats classified as pro-social (green) and non-pro-social (gray). The vertical red line represents the upper 95% confidence interval threshold obtained from the permutation analysis. **(C)** Correlation between partner weight index and social bias scores: we found a negative correlation between the social bias score and the normalized partner weight.

Overall, the above analysis, in which we pooled BR choices across all rats, showed that the rats' frequency of choosing the BR compartment was significantly above chance in the partner condition, but the effect was relatively small (55% BR choices on average). However, the preference for the BR compartment greatly varied across rats, i.e., some rats showed substantially higher preference for the BR alternative in the partner condition compared to the toy condition (Figure 3A—left), whereas others neither developed a preference for the BR alternative nor showed a condition-dependent choice pattern (Figure 3A—right). Thus, the overall mean fraction of BR choices may be diluted by the data from rats that did not display condition-dependent preferences. To determine the extent to which rats differed in their BR-preferences, we calculated a social bias score for each rat (SB, See Section Analysis) reflecting the percent difference in BR-choices in the partner compared to the toy condition. Thus, SB scores can be interpreted as estimates of how much more (or less) a rat preferred the BR-alternative in the partner relative to the toy condition. Furthermore, we compared each rats' SB score to a benchmark SB score distribution obtained through a bootstrapped permutation analysis (see Supplementary Data and Figure 3B; the red vertical line indicates the 95% confidence interval limit). We categorized all rats showing significantly higher SB scores than the upper confidence interval bound as pro-social (*N* = 29). All remaining animals were categorized as non-pro-social (*N* = 19). This analysis revealed a substantial degree of heterogeneity in preferences across rats, with SB scores ranging from −14.8 (14.8% more BR choices in the toy than in the partner condition) to 45.6 (45.6% more BR choices in the partner than in the toy condition).

Recently, body mass, already thought to reflect group hierarchy (Smith et al., 1994), has been shown to be a factor biasing rats' helping behavior toward lighter animals (Schneeberger et al., 2012). Therefore, we asked whether the individual differences in SB scores could be explained by the partners' individual weights. To this end, we correlated the SB scores with the normalized weight of the partners (the normalized weight parameter (see Section Analysis).

We found a negative correlation between normalized partner weight and SB scores (Figure 3C; *r* = −0.39, *p* = 0.006), suggesting that actors had a higher propensity to choose the BR alternative when paired with lighter partners. Interestingly, we also found a non-significant negative trend between average normalized actor weight and SB scores (*r* = −0.25, *p* = 0.08), fuelling the speculation that lighter actors may be more generous than heavier actors. We also computed the weight difference between individuals in each pair to investigate whether body mass differences between interacting animals could affect choice allocation. We found no significant correlation between weight difference and SB scores (Spearman rank correlation, *r* = 0.21, *p* = 0.14). Finally, we explored the possibility that the mere identity of the partner, independent of its body mass, could be related to the choice preferences of the actors. In batch two, each partner was paired with two different actors. This allowed us to test whether the two actors paired with a given partner usually showed similar, or divergent, BR-preferences. To this end, we quantified, for each partner, how many of its paired actors were classified as pro-social or non-pro-social and compared these observed counts to the number expected by chance. The observed categorization frequencies were not significantly different from the frequencies expected by chance (χ^2^ = 0.00, *p* = 1.00), suggesting that the mere identity of the partner did not trigger pro-social tendencies.

## Discussion

Using a novel, spatial rodent version of a Pro-social Choice Task (PCT; Silk et al., 2005), we tested whether rats showed non-costly helping behavior in a double T-Maze setup. Actor rats chose between two choice compartments yielding either just a reward for themselves, or an additional reward for a partner rat placed in an adjacent compartment. We contrasted the actors' percentage of BR choices in a partner condition with its BR choices in a toy condition, where the partner was an inanimate toy rat of similar shape, size and color. Importantly, the choice-reward payoff structure was identical across partner and toy conditions, i.e., rewards were delivered to the toy compartment with the same magnitude and delay as in the partner condition. Thus, any difference in choice distribution between both conditions would result from the influence of social context on decisions. If actors derive value from another rat's access to food, they should develop a preference for the BR alternative in the partner, but not in the toy condition. Our results confirmed that actors indeed revealed preferences for the alternative yielding food for their conspecific. Importantly, we show that the BR-preferences were contingent on the social element of the task, and not merely driven by secondary reinforcement properties of pellet delivery, such as the sound, smell or sight of rewards. In addition, we controlled for additional motives by always directing the partner to the compartment facing the actor's compartment, independent of the actor's choice; thus spatial proximity, social exploration motives, and approach/avoidance behavior are unlikely explanations of the actors' choices. Moreover, we found that BR-preferences quickly re-established after a repetition manipulation, suggesting that the observed behavior was stable over time. Finally, we found a negative correlation between the partner's weight and SB scores, indicating that actors had a higher propensity to choose the BR-alternative when the partner was light.

Although the frequency of the rats' choices of the BR alternative was significantly above chance, the average fraction of BR choices was relatively small (Figure 2). We argue that the reason for the relatively subtle overall preference for the BR-alternative lies in the great individual variability in our rats' BR preferences: while some rats showed a very clear and marked distinction between partner and toy conditions, increasing their BR-choices by >45% when paired with a real rat relative to a toy, others selected the BR alternative equally often in both conditions. Based on their individual sensitivity to the social context, we classified approximately 60% of our rats as pro-social, showing a significantly—sometimes considerably—larger preference for BR choices in the partner than in the toy condition, and roughly 40% of our rats as non-pro-social, showing no or little difference in BR choices between conditions.

Interestingly, in a study using a primate analog of the PCT, the authors found comparable levels of pro-social preferences, which they interpreted as evidence for variable spontaneous pro-social choice (Horner et al., 2011). Reports of pro-social tendencies in non-human primates are highly variable (Silk et al., 2005; Horner et al., 2011; Cronin, 2012), which might be due to the great individual differences in pro-social behavior, but may also result from the lack of standardization of the experimental designs used (House et al., 2014) and/or from socio-ecological differences between animal species, such as whether or not they engage in cooperative breeding (Burkart et al., 2014). Therefore, it is essential to establish a standardized social paradigm that allows for sound cross-species comparisons.

As mentioned above, we adopt a definition of pro-social choice promoted in the literature (Miller et al., 1991) as the preference for outcomes that produce a benefit for another individual. Importantly, to avoid any form of anthropomorphism and exercise interpretative caution (Morgan, 1903), we stress that this definition makes only very liberal claims about the underlying motives and mental mechanisms, and any behavior that increases the well-being of a conspecific would be labeled “pro-social” as long as that behavior happens in a genuinely social context and is driven by social motives, whatever they are. According to this definition, our rats' behavior qualifies as pro-social because the rats revealed a preference for outcomes that yielded food for another conspecific, and this preference was dependent on the social context (partner vs. toy).

Our study was designed to demonstrate the proof-of-principle that rats have pro-social preferences according to above definition, but, admittedly, it offers little direct insight into the individual motives driving pro-social behavior. So, what are the putative mental and neural mechanisms underlying pro-sociality? We propose that pro-social choices can be understood within a social reinforcement framework (Ruff and Fehr, 2014) where BR- and OR-outcomes are associated with social reinforcement value. That is, an actor's pro-social choice might be driven by (i) the appetitive consequence of positive social reinforcement, i.e., animals may seek—possibly rewarding—communication signals emitted by the partner after having received a reward (Kashtelyan et al., 2014; Willuhn et al., 2014), or the pleasure of eating rewards together (Barnett and Spencer, 1951). In addition (ii), the rats may be motivated by negative social reinforcement, i.e., they may avoid the—putatively aversive—distress signals emitted from the partner missing out on reward after selfish choices. Positive and negative social reinforcement are not mutually exclusive, but could act in concert to produce pro-social choice. Importantly, by controlling for non-social factors that could influence reinforcement learning in the context of this task, we maintain that preferences in the present task are genuinely social, i.e., dependent on social signals, such as the putative transmission and induction of affective states between actor and partner. Candidate substrates for a social transmission of affective states are ultrasonic vocalizations (USVs) which have been shown to reflect affective state in rats (Knutson et al., 1999; Wöhr et al., 2008; Takahashi et al., 2010). However, recent studies did not find evidence for a role for USVs in social transmission of fear (Pereira et al., 2012) or emotional contagion (Atsak et al., 2011). It is beyond the scope of this study to identify the actual communicative mechanisms driving pro-social behavior, but future studies should aim at isolating the motives underlying rodent pro-social choice.

The negative correlation between normalized partner weight and SB scores is also in line with the social reinforcement hypothesis: presumably, rats that are relatively hungrier might signal their state and/or respond more strongly to rewards bestowed on them, which might drive the actor's choice allocation toward the BR-compartment. Interestingly, pro-social behavior in non-human primates in possession of food seems to be fostered by begging (Gilby, 2006) and request (Warneken et al., 2007; Yamamoto et al., 2009, 2012; Melis et al., 2011) from conspecifics. However, recent results challenge this interpretation, as direct food sharing requests in chimpanzees did not trigger prosocial choice (Horner et al., 2011), nor did sympathy in great apes (Liebal et al., 2014). Interestingly, pro-social choice in long-tailed macaques has been shown to be hierarchy-dependent (Massen et al., 2011), i.e., dominant individuals grant food to their partners whereas subordinate ones withhold its access to their conspecifics (Massen et al., 2010). In rodents, recent findings showed that rats preferentially helped sated heavier, as well as lighter, hungrier partners (Schneeberger et al., 2012), thus suggesting a multi-factorial interaction between, at least, rank position and hunger state in helping behavior in rodents. Therefore, future studies using a PCT design should parametrically vary rank position and deprivation state in individual pairing to explore their role in rodent pro-social choice.

Interestingly, one recent study showed that rodent pro-social behavior was modulated by social experience (Ben-Ami Bartal et al., 2014), suggesting that potential pro-social preferences are influenced by social context. However, and importantly, our results also suggest that the partner's mere identity or behavior is not the single principal determinant of pro-social choice; it rather seems that the interaction between the partner's deprivation state and the actor's pro-social disposition is important for eliciting pro-social tendencies in the actor.

The current experimental design combines a series of advantages discussed elsewhere in the literature (see Cronin, 2012 for extensive discussion of this point). First, because pro-social choices were non-costly to the actor (Silk et al., 2005; Horner et al., 2011), we could de-confound pro-social motives from the rats' natural egoistic tendencies to maximize own payoff, which may have otherwise obscured any other-regarding considerations. Second, our task allowed the food to be hidden from the actors and partners during decision-making (Yamamoto and Tanaka, 2010; Horner et al., 2011), thus avoiding potential competitive or distractive influences on choice behavior. Third, partner rats could neither retaliate, nor return the favor, thus the actors' pro-social tendencies were not the result of strategic (tit-for-tat), reciprocal considerations. Finally, the toy condition was a crucial control manipulation: it allowed us to demonstrate that pro-social choice was directly contingent on the social component of the task, i.e., the presence of a real partner (Silk et al., 2005; Burkart et al., 2007), and not merely driven by secondary reinforcement mechanisms, such as the possibly motivational properties of the sensory features of the food rewards (sight, smell, dropping sound). Interestingly, animals in the first batch (but not the second batch) chose the BR alternative significantly below chance in the toy condition. One putative explanation to account for this counterintuitive result is that the rats showed a frustration effect, i.e., they assigned negative value to the pellets in the opposite non-social compartment that they could see and possibly also smell, but not access. Therefore, they might have avoided the delivery of such pellets by selecting the selfish option when paired with a non-social target. This explanation points toward multi-factorial effects: processes such as pellet delivery in the opposite compartment, or their inaccessibility in the non-social condition, might enter the decision process and reinforce subsequent behavior. Therefore, there might be multiple processes that promote or suppress the decision for the pro-social compartment. However, as this effect was not replicated in Batch 2, this explanation remains speculative.

In conclusion, we argue that the pro-social preferences of rats as demonstrated here result from social reward signals that should be further investigated in a social reinforcement learning framework. Our experimental design opens up the possibility for direct neurobiological and neuropharmacological interventions that might affect the processing, salience and/or evaluation of these putative social reward signals. For instance, it allows for behavioral, pharmacological and neurobiological interventions such as psychopharmacological manipulations of peptide- and hormone systems associated with pro-social behavior (Young et al., 1998), or manipulations of neural processes implicated in social behavior (Rushworth et al., 2007), as well as electrophysiological recordings (Buzsáki, 2004). Finally, the fact that non-primate animals show pro-social behavior in the absence of strategic, reciprocal or selfish motivations offers important insights into the evolution of pros-social behavior. Future studies could perform cross species investigations (Brosnan and de Waal, 2014) including the comparison of socio-ecological (Burkart et al., 2014) and methodological aspects of social behavior (Cronin, 2012) to reconcile diverging evidence on prosociality in the literature, and ultimately identify the factors driving its evolution.

## Author contributions

Julen Hernandez-Lallement designed and performed the research, analyzed the data and wrote the paper. Marijn van Wingerden designed the research and analyzed the data and wrote the paper. Christine Marx wrote the paper. Milan Srejic designed the research. Tobias Kalenscher acquired funds, designed the research, analyzed the data and wrote the paper. The study was supported by the Deutsche Forschungsgemeinschaft (DFG), grant n° KA2675/5-1.

### Conflict of interest statement

The authors declare that the research was conducted in the absence of any commercial or financial relationships that could be construed as a potential conflict of interest.

## Abbreviations

PCT: Pro-social Choice Task
BR: Both Reward
OR: Own Reward
ITI: Inter-Trial Interval.
