# Supplementary material for: Rats prefer mutual rewards in a prosocial choice task
Source: Front Neurosci. 2015 Jan 15;8:443. doi: 10.3389/fnins.2014.00443 (PMC4296215; doi:10.3389/fnins.2014.00443)
Supplement: Supplementary file 2 [file DataSheet1.DOCX]

**Rats act generously in a Pro-social Choice Task**

**Supplemental Information**

Julen Hernandez-Lallement*, Marijn van Wingerden, Christine Marx, Milan Srejic, Tobias Kalenscher

Comparative Psychology, Institute of Experimental Psychology, Heinrich-Heine University Düsseldorf, Universitätstrasse 1, 40225, Düsseldorf, Germany

***Correspondence:**

**Julen Hernandez Lallement, Comparative Psychology, Institute of Experimental Psychology,**

**Heinrich-Heine University Düsseldorf, Universitätsstrasse 1, 40225 Düsseldorf, Germany. e-mail: [julien.her@gmail.com](mailto:julien.her@gmail.com)**

**Supplemental Inventory**

1. **Supplemental Data**
2. **Supplemental Experimental Procedures**
3. **Subject and housing**
4. **Experimental design: Pre-Training**
5. **Analysis and statistics**

**3. Non-printable media file**

1. **Supplemental Data**

**No effect of condition order:** We found no significant differences in social bias scores between rats that started in the partner versus toy condition in batch 1 (Mann-Whitney U Test; U = 28.00; p = .67) and batch 2 (U = 94.00; p = .20).

1. **Supplemental Experimental Procedure**

In the first batch, two partner rats did not reach the behavioral criterion of performing ten consecutive trials per session in step 3 and were excluded from the experiment schedule.

1. **Subjects and housing**

Two batches (N = 20 and N = 48, respectively) of non-castrated male Long-Evans rats (*Janvier Labs,St. Berthevin, France*) were used, all animals weighed between 190-300g at the beginning of the experiment. Animals were housed in groups of four animals per cage, under an inverted 12:12 hours light - dark cycle (lights off at 07:00), in a temperature- (20 ± 2°C) and humidity-controlled (60%) colony room.

1. **Experimental design: Pre-Training**

All rats were habituated to the transport procedure between their stables and the testing setup, as well as the double T-maze setup for two sessions, after which they went through three steps of pre-training. During pre-training, rats entered each compartment five times (ten trials per session) in pseudo-randomized order.

Step 1: the experimenter let the rats enter one compartment and waited for the animals to find the reward location. Rats were then manually replaced in the starting box, and could enter the opposite compartment on a following trial. All animals reached optimal criterion (all pellets eaten within the five seconds after entering the compartment) after two sessions.

Step 2: After entering a choice compartment, rats were trapped by closing the compartment door, and replaced in the starting box five seconds after reward consumption. All rats reached criterion (all pellets eaten within the five seconds after entering the compartment) after two sessions.

Step 3: Forced trials were implemented as well as incremental delays (increasing step size of five seconds every session) between entering the compartment and reward delivery. Rats reached the behavioral criterion (same as step 2) for the final task after six sessions.

All rats accomplished all habituation and pre-training steps after twelve days of pre-training and started the Pro-social Choice Task. Before every session, partner and actor rats were allowed to physically interact for one minute.

1. **Analysis and statistics**

**Group categorization:** Animals were classified as pro-social if their bias score differed significantly from a bootstrapped reference permutation distribution. This permutation distribution of social bias scores consisted of N = 5000 draws of 10x2 sessions, with the percentage of BR choice of these sessions randomly assigned to partner and toy labels. For each of such draws, the resulting social bias score was calculated, generating a distribution of 5000 permuted social bias scores that followed a normal distribution. The 95% percentile of this distribution was selected as a benchmark social bias score, and subsequently the actual social bias score of each animal was then tested for significance against this condition-randomized social bias benchmark value.

**Statistics:**  Group level analyses were carried out using non-parametric testing: Wilcoxon signed rank test (paired samples), Mann-Whitney U Test (independent samples) and one sample Wilcoxon signed rank test (one-sample tests). Correlation analysis employed two-tailed non-parametric testing (Spearman’s rho). Analysis carried on the second batch used one-tailed testing because of the directionality of the hypothesized effect. Individual analysis used pooled data from both batches. The following significance levels were used: *p < .05 ; **p < .01 ; ***p < .001; ns = not significant. All statistical analyses were carried using IBM SPSS Statistics 20 and MatLab 2013a (The MathWorks).

1. **Non printable media file - Video. Experimental design and trial example:**

The video shows a typical training session in the partner condition for a rat in Batch 1. On that day, the BR side was set to the left actor's compartment. The actor rat (right on recording) performs two forced-choice trials followed by five free-choice trials. The partner rat's (left on recording) position is matched to the actor decision in every trial. Reward is delivered to the actor only or to both individuals for OR and BR choice, respectively. For demonstration purpose, only 7 trials are shown. A typical session consisted of 10 forced trials and 15 free trials. Pro-social side was randomized across sessions. Additionally, all rats went through a toy condition where the partner rat was replaced by a toy (not shown on recording). Frame per seconds have been increased to reduce file size. Thus, the video shows faster movement as in reality.
